# Global gene expression in neuroendocrine tumors from patients with the MEN1 syndrome

**DOI:** 10.1186/1476-4598-4-9

**Published:** 2005-02-03

**Authors:** William G Dilley, Somasundaram Kalyanaraman, Sulekha Verma, J Perren Cobb, Jason M Laramie, Terry C Lairmore

**Affiliations:** 1 Department of Surgery, Washington University School of Medicine, St. Louis, MO, USA; 2 John Cochran Veterans Administration Medical Center, St. Louis, MO, USA

## Abstract

**Background:**

Multiple Endocrine Neoplasia type 1 (MEN1, OMIM 131100) is an autosomal dominant disorder characterized by endocrine tumors of the parathyroids, pancreatic islets and pituitary. The disease is caused by the functional loss of the tumor suppressor protein menin, coded by the *MEN1 *gene. The protein sequence has no significant homology to known consensus motifs. *In vitro *studies have shown menin binding to JunD, Pem, Smad3, NF-kappaB, nm23H1, and RPA2 proteins. However, none of these binding studies have led to a convincing theory of how loss-of-menin leads to neoplasia.

**Results:**

Global gene expression studies on eight neuroendocrine tumors from MEN1 patients and 4 normal islet controls was performed utilizing Affymetrix U95Av2 chips. Overall hierarchical clustering placed all tumors in one group separate from the group of normal islets. Within the group of tumors, those of the same type were mostly clustered together. The clustering analysis also revealed 19 apoptosis-related genes that were under-expressed in the group of tumors. There were 193 genes that were increased/decreased by at least 2-fold in the tumors relative to the normal islets and that had a t-test significance value of p < = 0.005. Forty-five of these genes were increased and 148 were decreased in the tumors relative to the controls. One hundred and four of the genes could be classified as being involved in cell growth, cell death, or signal transduction. The results from 11 genes were selected for validation by quantitative RT-PCR. The average correlation coefficient was 0.655 (range 0.235–0.964).

**Conclusion:**

This is the first analysis of global gene expression in MEN1-associated neuroendocrine tumors. Many genes were identified which were differentially expressed in neuroendocrine tumors arising in patients with the MEN1 syndrome, as compared with normal human islet cells. The expression of a group of apoptosis-related genes was significantly suppressed, suggesting that these genes may play crucial roles in tumorigenesis in this syndrome. We identified a number of genes which are attractive candidates for further investigation into the mechanisms by which menin loss causes tumors in pancreatic islets. Of particular interest are: FGF9 which may stimulate the growth of prostate cancer, brain cancer and endometrium; and IER3 (IEX-1), PHLDA2 (TSS3), IAPP (amylin), and SST, all of which may play roles in apoptosis.

## Background

Multiple Endocrine Neoplasia type 1 (MEN1, OMIM 131100) is an autosomal dominant disorder characterized by endocrine tumors of parathyroid, pancreatic islets and pituitary [1]. The prevalence of MEN1 is estimated to be 2–10 per 100,000 [2]. Based on loss of heterozygosity in tumors and Knudson's "two-hit" hypothesis, the *MEN1 *gene was classified as a tumor suppressor [2,3] and the gene was isolated in 1997 by positional cloning [4]. The *MEN1 *gene spans 9 kb of the genome, is comprised of 10 exons, and codes for a 610 amino acid protein termed menin [4]. More than 300 independent germline and somatic mutations have been identified [5]. Recently, five new germline mutations which affect splicing of pre-mRNA transcribed from *MEN1 *gene were identified in our laboratory [6]. The nature of all the disease-inducing mutations points to a loss of function of menin, which is characteristic of a tumor suppressor. Database analysis of menin protein sequence reveals no significant homology to known consensus protein motifs. Menin is widely expressed in both endocrine and non-endocrine tissues [4]. Menin is primarily localized in the nucleus and contains two nuclear localization signal sequences near the carboxyl terminus of the protein [7].

Studies on the function of menin have not yielded a clear picture as to the role of menin as a tumor suppressor; however, the results of these studies suggest some interesting possibilities. Two groups [8,9], based on yeast two-hybrid screening of a human adult brain library, reported that menin interacts with JunD (a member of the AP-1 transcription factor family) and represses JunD mediated transcription. Recently, Agarawal *et al*[10] reported that when JunD loses its association with menin it becomes a growth promoter rather than a growth suppressor. Other reports suggest some relevance of the menin-JunD interaction. JunD null male mice exhibit impaired spermatogenesis [11]. In postnatal mouse, *Men1 *was found to be expressed in testis (spermatogonia) at high levels [12]. Lemmens *et al *[13] by screening a 12.5 dpc mouse embryo library with menin, identified a homeobox-containing mouse protein, Pem. Interestingly, both menin and Pem showed a very similar pattern of expression, especially in testis and Sertoli cells. These findings along with the fact that some MEN1 patients have idiopathic oligospermia and non-motility of spermatozoa [14] suggest that menin-JunD and menin-PEM interactions may play a vital role in spermatogenesis. Kaji *et al *[15] observed that menin interacts with Smad3 and inactivation of the former blocks transforming growth factor beta (TGF-β) signaling in pituitary tumor derived cell lines. Recently, two more menin interacting proteins, NF-kappa B [16] and a putative tumor metastasis suppressor nm23 [17] have been identified. Interactions among AP-1 family members, Smad proteins and NF-kappa B have been documented [18-21] and such cross talk among signaling pathways is not uncommon.

Despite the above studies, a clear consensus of the molecular mechanisms leading to neoplasia, following the loss of menin, has not emerged. Very little is known about the gene expression changes in human neuroendocrine tumors following the loss of menin. Global gene expression analyses, using cDNA microarrays, have been used to classify other human tumors into clinically distinct categories [22-26]. Wu [27] has discussed the mathematical and statistical considerations for the use of DNA microarrays to identify genes of specific interest, and Harkin [28] has used expression profiling to identify downstream transcriptional targets of the BRCA1 tumor suppressor gene. Our objective was to identify genes that might be directly or indirectly over or under-expressed as a consequence of loss of menin expression.

## Results

### Patients and Controls

Eight neuroendocrine tumors from six MEN1 patients were included in this study. The patient ages were 19, 22, 42, 51, 57, and 57 years at the time of surgery (Table 1). One was female, and five were male. Two of the patients had clinical and laboratory findings consistent with insulinoma. Three tumors were analyzed from one of these patients. One patient had findings consistent with VIP-oma (vasoactive intestinal polypeptide secreting tumor). Two patients, with no specific symptoms, had non-functioning or pancreatic polypeptide secreting tumors. One patient had symptoms of gastrinoma from a duodenal tumor (not used for this analysis). A pancreatic tumor from this patient, found incidentally, was used in this study. Pathological examination of tumors from the 6 patients resulted in the classification of 3 insulinomas, 3 neuroendocrine tumors, 1 VIP-oma and 1 glucagonoma. The ages of the individuals donating normal pancreatic islets were 42, 52(2), and 56 years. Two were female, and two were male.

**Table 1:** Characteristics of patients and normal subjects.

**Pt.#**	**T #**	**Age**	**Sex**	**Clinical**	**LN Mets**	**T Vol. (ml)**	**Menin Defect [6]**
1	1	19	F	Insulinoma	0/1	8.28	Large Deletion, exon 1 & 2
2	2	42	M	Neuroendocrine Tumor	0/14	18.75	Nonesense Mutation, exon 7
6	6	60	M	VIP-oma	1/16	288	8 bp Deletion, exon 5
7	7	51	M	Neuroendocrine Tumor	2/30	3.75	2 bp Deletion, exon 2
8	8–10	22	M	Insulinoma	2/8	6.9	2 bp Deletion, exon 2
11	11	57	M	Gastrinoma	1/1	0.5	4 bp Deletion, exon 3
N1	N1	52	M	Normal	NA	NA	NA
N2	N2	56	F	Normal	NA	NA	NA
N3	N3	52	F	Normal	NA	NA	NA
N4	N4	42	M	Normal	NA	NA	NA

### Quality of Hybridization

**Figure 1:**
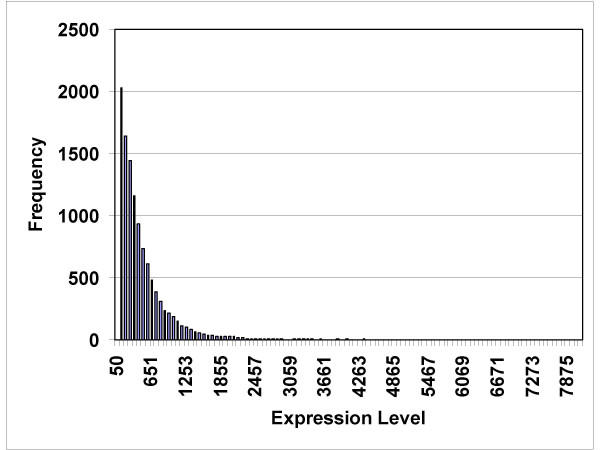
Histogram showing the frequency of genes being expressed at levels between 50 and 7875 (arbitrary expression units).

The RNA isolated from 8 tumor specimens (6 patients) and 4 normal islet preparations was of acceptable quality for hybridization, as determined by preliminary small hybridizations on test chips. The dChip computer program returned data concerning the percent of genes judged to be present, and the percent of single and array outlier events (Table 2). The expression data from one normal islet preparation had 5.94% array outliers, which prompted dChip to issue a warning (a warning indicates more than 5% array outliers detected). However, since we had only four normal specimens, we elected to include all four in our analysis. The average level of gene expression was computed for each gene (Figure 1). The average gene expression level for all genes followed an exponentially decreasing pattern; the greatest number of genes had expression values less than 100, and only a few genes had expression levels greater than 4000.

**Table 2:** Overall statistics on the quality of each the processed GeneChips. One chip was used for each tumor/normal specimen. The "Median Intensity" refers to the overall brightness of the fluorescence of the genes. The "Present Call" refers to whether the gene was "present" or "absent".

**Chip Name**	**Median Intensity**	**Present Call (%)**	**Array outlier %**	**Single outlier %**	**Warning**
T1	170	49.4	1.12	0.11	
T2	107	46.2	1.54	0.15	
T6	160	51.4	1.16	0.12	
T7	132	47.7	0.50	0.08	
T8	158	51.0	0.59	0.10	
T9	114	48.9	0.66	0.10	
T10	158	50.6	0.42	0.07	
T11	121	46.1	3.34	0.30	
N1	142	48.4	2.65	0.26	
N2	179	49.7	2.72	0.24	
N3	75	48.3	3.38	0.31	
N4	73	33.2	9.50	0.63	*

### Overall Consistency of Gene Expression

**Figure 2:**
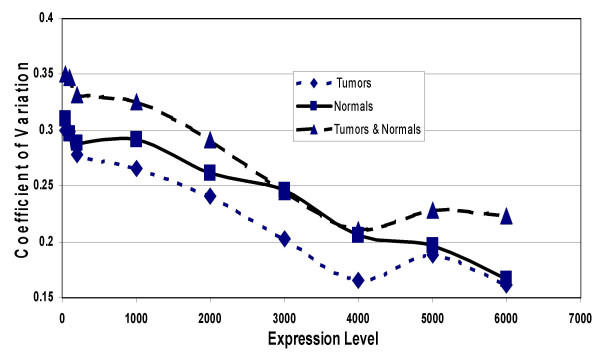
Coefficient of variation (CV) of genes being expressed at levels between 50 and 6000. For each gene expressed at an average level of 50 or above, the CV was computed for the group of 8 tumors, for the group of 4 normals, and for the group of all 12 tumors and normals. As the lower limit of expression was increased, the number of genes represented in the CV decreased: there were 12,000 genes with expression levels of 50 or more, but only a few genes with expression levels of 6,500 or more.

Average expression and standard deviation was computed for each gene in both the group of 4 normal islets, and the group of 8 islet tumors and expressed as the coefficient of variation (CV). Genes with average expression levels less than 50 were excluded from this analysis. Figure 2 shows that the average (11,416 genes and expressed sequences) CV in the group of 8 tumors was 30%. There was a linear regression of CV values as the average minimum expression level of the genes increased. Genes with an average minimum expression level of 7000 or more had an average CV level of 12.7%. The analysis of genes expressed in the normal islets gave similar results. However, when the tumors were combined with the normals, the CV was higher than either group alone. This was caused by the true differences in gene expression levels between the tumors and the normals.

### Clustering

**Figure 3:**
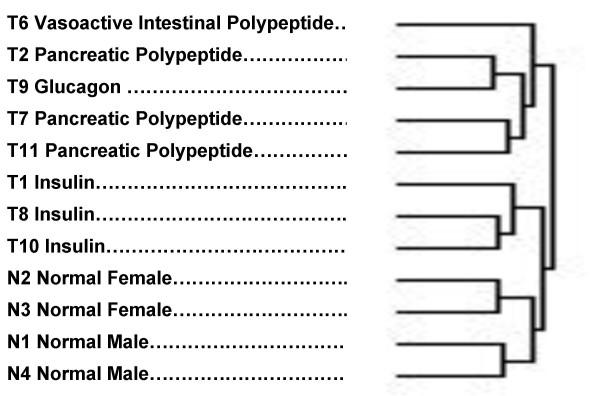
Clustering of tumors and normals according to overall gene expression patterns. The predominant type of hormone expression (Table 3) is noted for each tumor/normal specimen.

The experimental groups were clustered (figure 3) using a hierarchical clustering procedure [29,30]. This cluster was based on the inclusion of all genes which had 33% to 67% of "present" calls made by the GeneChip software. The assignment of tumor type was made on the basis of principal hormone messenger RNA levels that were consistent with the clinical and biochemical findings (Table 3). The principal bifurcation in the clustering occurred between the group that included the normal specimens and the three tumors with a predominance of insulin expression, on one hand, and the other tumor types on the other. The four normal islet preparations clustered together, separate from the tumors. Among the normal islets, the females clustered separately from the males. Among the tumors, all 3 insulinomas clustered together, separate from the VIP-oma, the glucagonoma and the PP-omas (pancreatic polypeptide producing tumors). It is also interesting that all the specimens clustered in a pattern of increasing malignancy going from normal at the bottom of the cluster to most malignant at the top.

**Table 3:** Gene expression levels of islet hormone mRNAs in tumors and normals. VIP: Vasoactive intestinal polypeptide; PP: Pancreatic polypeptide.

	**T1**	**T2**	**T6**	**T7**	**T8**	**T9**	**T10**	**T11**	**N1**	**N2**	**N3**	**N4**
**pre-Gastrin**	864	530	678	392	600	383	209	395	1036	775	28	1192
**Insulin**	9990	13	179	401	10195	240	8971	1831	10010	9752	9580	8158
**Glucagon**	10	6482	2783	1198	10	8370	10	10	9037	8425	9043	7800
**VIP**	351	278	10243	374	334	276	362	202	806	436	334	389
**PP**	246	7257	577	5845	70	1805	211	8895	1897	7605	3598	1177

**Figure 4:**
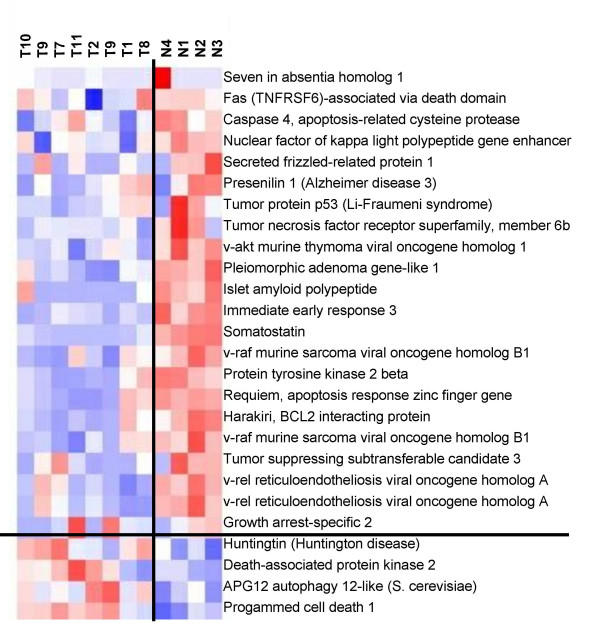
Clustering of apoptosis-related genes in tumors (T) and normals (N). Pink indicates strong, white indicates moderate, and blue indicates weak expression.

The genes were also clustered by the dChip software. A group of apoptosis-related genes was identified whose expression was significantly correlated with the Tumor/Normal assignment of the data. Twenty-four apoptosis-related genes represented by 26 different Affymetrix probes were identified in the overall hierarchical clustering. Nineteen of these genes were more highly expressed in the normal islets than in the islet tumors (Figure 4). Eighteen of the nineteen under expressed genes in the set of tumors had t-test p values (tumor vs. normal) <= 0.037. All five of the apoptosis-related genes, that were more highly expressed in the tumors, had t-test p values >0.05

### Evaluation of Student's t-test

Since the Student's t-test was designed to compare only one parameter in two populations, the simultaneous measurement of multiple genes might lead to an excessive number of false positives. In order to empirically determine the potential false positive rate, we started with 923 genes which had a p value <=.05 and repeatedly scrambled the individual tests into groups 4 and 8 and then performed new t-tests. The average number of genes having a p value = < .05 in 20 such scrambles was 51 (5.5% of 923 genes). This was only slightly more than the 46 genes expected (0.05 × 923). We therefore concluded that there was little chance of excess false positives in repeatedly using the Student's t-test.

### Hormone Expression Profiles

In order to obtain a better picture of the nature of the tumors and normal islets in this study, the expression levels of the principal hormone RNA of pancreatic islets was examined (Table 3). Tumors 1, 8, and 10 had high levels of insulin expression and came from patients with the clinical diagnosis of insulinoma. Tumor 6 had high levels of VIP and came from a patient with the clinical syndrome of VIP-oma. Tumors 2 and 7 had high levels of pancreatic polypeptide, and came from patients with only a diagnosis of neuroendocrine tumor. Tumor 9, which came from a patient with a clinical diagnosis of insulinoma had a high level of glucagon expression; the clinical diagnosis was apparently due to the other tumor (#8) which did have a high level of insulin expression. One other apparent discrepancy between the clinical diagnosis and hormone expression profile occurred with tumor 11, which had high a level of glucagon expression. This patient had an additional duodenal tumor that was responsible for the gastrin secretion and the clinical diagnosis. All the normal islet preparations had high levels of insulin and glucagon expression, as expected.

### Comparison of tumor and normal gene expression

The reporting of differentially expressed genes was restricted to those in which the absolute ratio of Tumor to Normal was greater than or equal to 2, and which had a Student's t-test p value of less than or equal to .005. There were 193 genes that met the criteria. Expressed sequences with no known protein product were not included. There were 45 genes that were increased in the tumors relative to the normals, and 148 genes that were decreased. The fold-change in expression values ranged from +179 to -449. Genes were assigned to functional categories based on the Gene Ontology Consortium assignments . There were 16 genes related to cell growth, 13 genes related to signal transduction, and 16 genes related to other functions which were increased in the group of tumors relative to the group of normal islets (Table 4). There were 44 genes related to cell growth, 10 related to cell death, 10 related to embryogenesis, 5 related to nucleic acid binding, 21 related to cell signaling, and 58 related to other functions in the group of genes which were decreased in the islet tumors relative to the controls (Tables 5, 6, 7, 8).

**Table 4:** Genes significantly increased in tumors.

**GeneBank Accession**	**Gene**	**Symbol**	**Normal Mean**	**Tumor Mean**	**Fold Change**	**P value**
	**Cell Growth/Cycle**					
X16323	hepatocyte growth factor	HGF	11	116	10.77	0.003305
AB017642	oxidative-stress responsive 1	OSR1	58	428	7.41	0.000819
AL078641	phorbolin-like protein	APOBEC3G	15	92	6.21	0.000158
L17128	gamma-glutamyl carboxylase	GGCX	64	346	5.37	0.000018
D21089	xeroderma pigmentosum, complementation group C	XPC	292	1278	4.38	0.000284
AL050223	vesicle-associated membrane protein 2	VAMP2	360	1533	4.26	0.002196
D38145	prostaglandin I2 synthase	PTGIS	29	121	4.09	0.000448
AF092563	structural maintenance of chromosomes 2-like 1	SMC2L1	58	185	3.21	0.002352
AF006087	actin related protein 2/3 complex, subunit 4	ARPC4	292	865	2.96	0.000565
AC004537	inhibitor of growth family, member 3	ING3	46	114	2.47	0.003976
AF013168	tuberous sclerosis 1	TSC1	35	86	2.45	0.001232
AJ236876	ADP-ribosyltransferase polymerase)-like 2	ADPRTL2	32	76	2.34	0.003874
	**Cell Death/Apoptosis**					
D38435	postmeiotic segregation increased 2-like	PMS2L1	74	193	2.6	0.002976
M61906	phosphoinositide-3-kinase, regulatory subunit	PIK3R1	43	104	2.4	0.004387
	**Signal Transduction**					
U26710	Cas-Br-M ectropic retroviral transforming sequence b	CBLB	21	177	8.4	0.000082
AB010414	guanine nucleotide binding protein, gamma 7	GNG7	59	334	5.68	0.003835
U59913	mothers against decapentaplegic homolog 5	MADH5	14	73	5.22	0.004731
AB004922	Homo sapiens gene for Smad 3	MADH3	93	443	4.76	0.001024
L11672	zinc finger protein 91	ZNF91	428	2007	4.69	0.000376
D14838	fibroblast growth factor 9	FGF9	27	108	3.97	0.000752
W27899	member RAS oncogene family	RAB6B	68	232	3.43	0.00501
U48251	protein kinase C binding protein 1	PRKCBP1	40	127	3.18	0.001999
U90268	cerebral cavernous malformations 1	CCM1	53	151	2.87	0.004392
AL050275	cysteine rich with EGF-like domains	CRELD1	195	543	2.79	0.000828
AB014600	SIN3 homolog B, transcriptional regulator	SIN3B	177	425	2.39	0.001924
M27691	cAMP responsive element binding protein 1	CREB1	107	229	2.15	0.003559
U85245	phosphatidylinositol-4-phosphate 5-kinase, type II, beta	PIP5K2B	244	518	2.12	0.000441
W25793	ring finger protein 3	RNF3	163	326	2	0.004947
	**Nucleic Acid Binding**					
D50912	RNA binding motif protein 10	RBM10	96	443	4.6	0.001925
U41315	makorin, ring finger protein, 4	MKRN4	404	808	2	0.000262
	**Ligand Binding**					
X67155	kinesin-like 5	KIF23	64	368	5.76	0.001584
AB028985	ATP-binding cassette, sub-family A, member 2	ABC1	65	262	4.04	0.001234
Z48482	matrix metalloproteinase 15	MMP15	139	495	3.56	0.003946
	**Enzyme**					
X13794	lactate dehydrogenase B	LDHB	396	1606	4.05	0.000845
X15334	creatine kinase, brain	CKB	939	2083	2.22	0.002008
X60708	dipeptidylpeptidase IV	DPP4	133	291	2.19	0.000697
AC004381	SA homolog	SAH	283	599	2.11	0.000168
AF000416	exostoses-like 2	EXTL2	134	271	2.02	0.001314
	**Embryogenesis**					
U48437	amyloid beta precursor-like protein 1	APLP1	851	2433	2.86	0.001043
U66406	ephrin-B3	EFNB3	168	438	2.6	0.00309
D50840	UDP-glucose ceramide glucosyltransferase	UGCG	85	211	2.5	0.002554
	**Other/Unknown**					
L48215	hemoglobin, beta	HBB	12	2099	178.78	0.001299
J00153	hemoglobin, alpha 1	HBA1	15	1249	82.25	0.001889
U30521	P311 protein	C5orf13	157	453	2.88	0.001431
AB011169	similar to S. cerevisiae SSM4	TEB4	140	300	2.15	0.00154
AL031432	GCIP-interacting protein	P29	99	198	2	0.002036

**Table 5:** Genes significantly decreased in tumors.

**GeneBank Accession**	**Gene Description**	**Symbol**	**Normal Mean**	**Tumor Mean**	**Fold Change**	**P value**
	**Cell Growth/Division**					
D17291	regenerating protein I beta	REG1B	6286	13	-499.46	0.000095
X67318	carboxypeptidase A1	CPA1	3928	121	-32.57	0.003205
AI763065	regenerating islet-derived 1 alpha	REG1A	5641	334	-16.88	0.000001
D29990	solute carrier family 7, member 2	SLC7A2	2988	445	-6.72	0.002204
AB017430	kinesin-like 4	KIFF22	1223	316	-3.87	0.000177
Z25884	chloride channel 1	CLCN1	2511	655	-3.84	0.00013
X81438	amphiphysin	AMPH	2686	752	-3.57	0.000002
L03785	myosin, light polypeptide 5	MYL5	207	59	-3.51	0.000233
W28062	guanine nucleotide-exch. Prot. 2	ARFGEF2	66	19	-3.46	0.003602
X52486	uracil-DNA glycosylase 2	UNG2	2555	756	-3.38	0.000514
M81933	cell division cycle 25A	CDC25A	312	96	-3.25	0.000005
M69136	chymase 1	CMA1	360	115	-3.13	0.004413
U90543	butyrophilin	BTN2A1	685	226	-3.04	0.000023
X69086	utrophin	UTRN	1325	457	-2.90	0.000011
AF039241	histone deacetylase 5	HDAC5	1124	393	-2.86	0.000319
U49392	allograft inflammatory factor 1	AIF1	165	58	-2.82	0.000105
U81992	pleiomorphic adenoma gene-like 1	PLAGL1	330	118	-2.80	0.004717
L26336	heat shock 70kD protein 2	HSPA2	90	32	-2.79	0.000689
F27891	cytochrome c oxidase subunit VIa	COX6A2	872	313	-2.79	0.000342
D87673	heat shock transcription factor 4	HSF4	1964	721	-2.73	0.000453
X97795	RAD54-like	RAD54L	392	144	-2.72	0.001345
X92689	UDP-N-acetyl-alpha-D-galactosamine	GALNT3	80	32	-2.50	0.000243
Y08683	carnitine palmitoyltransferase I	CPT1B	1038	420	-2.47	0.000573
U40622	X-ray repair complementing defective repair 4	XRCC4	177	72	-2.45	0.000678
U64315	excision repair, complementation group 4	ERCC4	2122	868	-2.44	0.000045
AB020337	beta 1,3-galactosyltransferase	B3GALT5	1489	635	-2.34	0.002613
U40152	origin recognition complex	ORC1L	3671	1702	-2.16	0.001425
M10943	metallothionein 1F	MT1F	5691	2653	-2.14	0.001707
X79882	major vault protein	MVP	758	376	-2.02	0.001719
AF035960	transglutaminase 5	TGM5	3097	1542	-2.01	0.002951
						
	**Cell Death/Apoptosis**					
S81914	immediate early response 3	IER3	2209	480	-4.60	0.000307
D80007	programmed cell death 11	PDCD11	457	129	-3.55	0.002358
AF013956	chromobox homolog 4	CBX4	1599	492	-3.25	0.00034
U33284	protein tyrosine kinase 2 beta	PTK2B	693	237	-2.93	0.000763
U90919	likely partner of ARF1	APA1	2687	1021	-2.63	0.000015
X57110	Cas-Br-M retroviral transforming	CBL	1889	784	-2.41	0.000033
AL050161	pro-oncosis receptor	PORIMIN	1178	497	-2.37	0.00031
U40380	presenilin 1	PSEN1	1301	569	-2.29	0.00012
D83699	harakiri, BCL2 interacting protein	HRK	768	338	-2.27	0.001321
U07563	v-abl viral oncogene homolog 1	ABL1	1415	631	-2.24	0.000248
M95712	v-raf oncogene homolog B1	BRAF	338	157	-2.16	0.004207
M16441	lymphotoxin alpha	LTA	2106	985	-2.14	0.000239
AF035444	pleckstrin homology-like domain, family A, member 2	PHLDA2	334	166	-2.01	0.001759

**Table 6:** Genes significantly decreased in tumors (continued).

**GeneBank Accession**	**Gene Description**	**Symbol**	**Normal Mean**	**Tumor Mean**	**Fold Change**	**P value**
	**Signal Transduction**					
J00306	somatostatin	SST	7701	284	-27.09	0
AI636761	somatostatin	SST	7224	598	-12.09	0.000001
AB011143	GRB2-associated binding protein 2	GAB2	2237	402	-5.57	0.001816
M93056	serine (or cysteine) proteinase inhibitor	SERPINB1	505	105	-4.80	0.004637
X68830	islet amyloid polypeptide	IAPP	2231	477	-4.68	0.001221
AB029014	RAB6 interacting protein 1	RAB6IP1	824	181	-4.56	0.000155
AI198311	neuropeptide Y	NPY	610	154	-3.96	0.004817
M28210	member RAS oncogene family	RAB3A	2566	672	-3.82	0.000048
J04040	glucagon	GCG	8620	2351	-3.67	0.000396
AF030335	purinergic receptor P2Y	P2RY11	2314	680	-3.40	0.000058
M29335	major histocompatibility complex	HLA-DOA	906	268	-3.39	0.00159
L38517	Indian hedgehog homolog	IHH	3013	897	-3.36	0.000055
U95367	gamma-aminobutyric acid A receptor, pi	GABRP	668	202	-3.30	0.000837
W28558	pleiotropic regulator 1	PLRG1	704	216	-3.26	0.000068
L08485	gamma-aminobutyric acid A receptor, alpha 5	GABRA5	342	107	-3.20	0.000336
AF004231	leukocyte immunoglobulin-like receptor	LILRB2	93	30	-3.08	0.001105
AF055033	insulin-like growth factor binding protein 5	IGFBP5	126	43	-2.96	0.000257
AJ010119	ribosomal protein S6 kinase	RPS6KA4	1532	522	-2.94	0.000201
U46194	Human renal cell carcinoma antigen	RAGE	2057	754	-2.73	0.000324
L13858	son of sevenless homolog 2	SOS2	964	354	-2.72	0.000268
Z29572	tumor necrosis factor receptor superfamily	TNFRSF17	184	68	-2.69	0.000178
U01134	fms-related tyrosine kinase 1	FLT1	910	379	-2.40	0.003257
D78156	RAS p21 protein activator 2	RASA2	327	144	-2.26	0.002332
U77783	glutamate receptor	GRIN2D	518	240	-2.15	0.001379
D49394	5-hydroxytryptamine receptor 3A	HTR3A	197	98	-2.02	0.002493
						
	**Nucleic Acid Binding**					
Z30425	nuclear receptor subfamily 1, group I, member 3	NR1I3	1008	356	-2.83	0.000329
U18760	nuclear factor I/X	NFIX	5796	2216	-2.62	0.000711
AI223140	purine-rich element binding protein A	PURA	1137	506	-2.25	0.002448
AF015950	telomerase reverse transcriptase	TERT	561	255	-2.20	0.002839
U40462	zinc finger protein, subfamily 1A, 1	ZNFN1A1	662	308	-2.15	0.001171
Z93930	X-box binding protein 1	XBP1	2223	1061	-2.09	0.000277
AB019410	PET112-like	PET112A	1422	707	-2.01	0.001309
						
	**Ligand Binding**					
X00129	retinol binding protein 4, plasma	RBP4	1517	68	-22.27	0.004809
AJ223317	sarcosine dehydrogenase	SARDH	3844	1069	-3.60	0.000085
AB017494	LCAT-like lysophospholipase	LYPLA3	906	326	-2.78	0.001131
U78735	ATP-binding cassette, sub-family A, member 3	ABCA3	1914	706	-2.71	0.000288
AF026488	spectrin, beta, non-erythrocytic 2	SPTBN2	1604	671	-2.39	0.00005
U83659	ATP-binding cassette, sub-family C, member 3	ABCC3	1287	551	-2.34	0.00244
R93527	metallothionein 1H	MT1H	5093	2196	-2.32	0.002937
AA586894	S100 calcium binding protein A7	S100A7	507	221	-2.29	0.000537
U91329	kinesin family member 1C	KIF1C	2981	1484	-2.01	0.000518

**Table 7:** Genes significantly decreased in tumors (continued).

**GeneChip Accession**	**Gene Description**	**Symbol**	**Normal Mean**	**Tumor Mean**	**Fold Change**	**P value**
	**Enzyme**					
M81057	carboxypeptidase B1	CPB1	4534	79	-57.09	0.001106
X71345	protease, serine, 4	PRSS3	3859	76	-51.11	0.004102
X01683	serine (or cysteine) proteinase inhibitor, clade A	SERPINA1	2550	74	-34.64	0.004833
M24400	chymotrypsinogen B1	CTRB1	5158	207	-24.95	0.001744
M18700	elastase 3A, pancreatic	ELA3A	7058	384	-18.37	0.000009
U66061	protease, serine, 1	PRSS1	7291	645	-11.31	0.000047
L22524	matrix metalloproteinase 7	MMP7	595	54	-11.03	0.002591
AI655458	5-oxoprolinase (ATP-hydrolysing)	OPLAH	446	99	-4.52	0.004072
H94881	FXYD domain-containing ion transport regulator 2	FXYD2	3116	708	-4.40	0.000539
AL021026	flavin containing monooxygenase 2	FMO2	905	215	-4.21	0.000804
AC005525	plasminogen activator, urokinase receptor	PLAUR	1779	566	-3.14	0.000031
U40370	phosphodiesterase 1A, calmodulin-dependent	PDE1A	268	89	-3.03	0.004023
R90942	sialyltransferase 7D	SIAT7D	3148	1052	-2.99	0.002319
M84472	hydroxysteroid (17-beta) dehydrogenase 1	HSD17B1	1196	440	-2.72	0.000991
X55988	ribonuclease, RNase A family, 2	RNASE2	480	203	-2.36	0.001314
AB003151	carbonyl reductase 1	CBR1	4538	1945	-2.33	0.000511
X08020	glutathione S-transferase M1	GSTM1	2766	1376	-2.01	0.000519
						
	**Embryogenesis**					
U15979	delta-like homolog	SIGLEC5	3384	402	-8.41	0.002927
M60094	H1 histone family, member T	HIST1H1T	976	230	-4.23	0.001639
U50330	bone morphogenetic protein 1	BMP1	3298	973	-3.39	0.001637
M74297	homeo box A4	HOXA4	501	176	-2.85	0.000477
AJ011785	sine oculis homeobox homolog 6	SIX6	530	190	-2.79	0.000286
U66198	fibroblast growth factor 13	FGF13	191	73	-2.61	0.001068
D31897	double C2-like domains, alpha	DOC2A	1151	451	-2.55	0.000068
U12472	glutathione S-transferase pi	GSTP1	3122	1524	-2.05	0.000237
						
	**Transcription**					
AL049228	pleckstrin homology domain interacting protein	PHIP	257	33	-7.69	0.000782
M27878	zinc finger protein 84	ZNF84	54	15	-3.64	0.001108
U77629	achaete-scute complex-like 2	ASCL2	438	184	-2.38	0.000058
D50495	transcription elongation factor A, 2	TCEA2	1330	595	-2.23	0.000019
U49857	transcriptional activator of the c-fos promoter	CROC4	542	259	-2.09	0.003894

**Table 8:** Genes significantly decreased in tumors (continued).

**GeneBank Accession**	**Gene Description**	**Symbol**	**Normal Mean**	**Tumor Mean**	**Fold Change**	**P value**
	**Other/Undefined**					
X72475	immunoglobulin kappa constant	IGKC	1409	276	-5.11	0.000111
D17570	zona pellucida binding protein	ZPBP	355	71	-5.02	0.001107
M90657	transmembrane 4 superfamily member 1	TM4SF1	592	141	-4.20	0.004537
AF063308	mitotic spindle coiled-coil related protein	SPAG5	2015	502	-4.01	0.000588
U66059	T cell receptor beta locus	TRB@	3022	779	-3.88	0.000266
AL022165	carbohydrate sulfotransferase 7	CHST7	359	94	-3.82	0.001738
U10694	melanoma antigen, family A, 9	MAGEA9	1039	272	-3.82	0.000067
M73255	vascular cell adhesion molecule 1	VCAM1	80	22	-3.66	0.004179
U47926	leprecan-like 2 protein	LEPREL2	1003	319	-3.15	0.00013
L05424	CD44 antigen	CD44	1439	471	-3.05	0.001361
AI445461	transmembrane 4 superfamily member 1	TM4SF1	463	161	-2.88	0.002911
AF010310	proline oxidase homolog	PRODH	1194	421	-2.84	0.000005
AF000991	testis-specific transcript, Y-linked 2	TTTY2	700	254	-2.76	0.000542
X57522	transporter 1, ATP-binding cassette, sub-family B	TAP1	781	287	-2.72	0.000971
AA314825	trefoil factor 1	TFF1	1657	616	-2.69	0.000011
AB020880	squamous cell carcinoma antigen	SART3	3228	1224	-2.64	0.000135
AF040707	homologous to yeast nitrogen permease	NPR2L	1131	437	-2.59	0.001537
U47292	trefoil factor 2	TFF2	359	141	-2.54	0.000684
X69398	CD47 antigen	CD47	350	144	-2.42	0.000853
U27331	fucosyltransferase 6	FUT6	1105	473	-2.34	0.000872
AI827730	cyclin M2	CNNM2	5863	2535	-2.31	0.000484
U05255	glycophorin B	GYPB	1606	717	-2.24	0.00013
M34428	pvt-1 oncogene homolog, MYC activator	PVT1	1231	550	-2.24	0.004423
U86759	netrin 2-like	NTN2L	2039	937	-2.18	0.000204
D90278	CEA-related cell adhesion molecule 3	CEACAM3	4388	2024	-2.17	0.000902
L40400	ZAP3 protein	ZAP3	1549	719	-2.15	0.000776
U48224	beaded filament structural protein 2, phakinin	BFSP2	568	271	-2.10	0.000166
AI138834	deltex homolog 2	DTX2	311	148	-2.10	0.000687
M13755	interferon-stimulated protein, 15 kDa	G1P2	1507	741	-2.03	0.001157
X52228	mucin 1, transmembrane	MUC1	1523	756	-2.02	0.001707

### Validation of GeneChip Data with Quantitative RT-PCR

**Figure 5:**
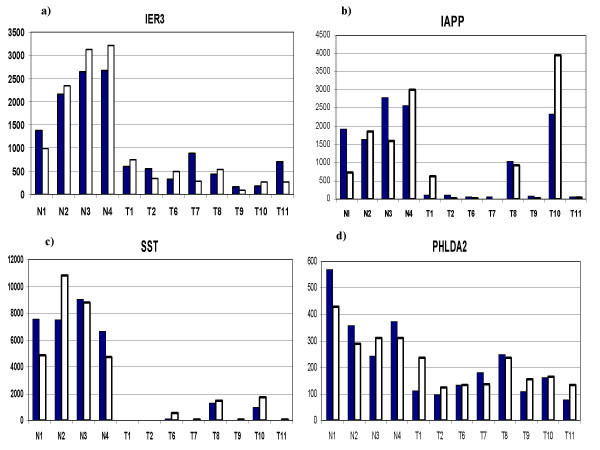
The expression levels of 4 apoptosis-related genes are shown by GeneChip and quantitative RT-PCR: a) IER3; b) IAPP; c) SST; d) PHLDA2. Normals (N) and tumors (T) are shown. Solid bars represent GeneChip and open bars represent Q-PCR results.

**Figure 6:**
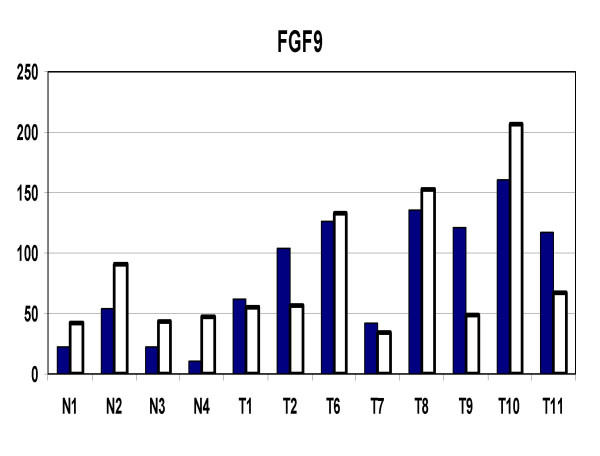
FGF9 expression levels in tumors (T) and normals (N) by GeneChip and quantitative RT-PCR. Solid bars represent GeneChip and open bars represent Q-PCR results.

In order to evaluate how accurately the GeneChip data was representing actual gene expression levels, eleven genes were tested with quantitative RT-PCR (Q-PCR). The results are shown in Table 9. The correlation coefficients ranged from 0.964 to 0.235 with an average of 0.655. The lower correlation coefficients were associated with genes with larger numbers of exons. There was some association of low correlation with low average numerical expression values. The lowest correlations were associated with very faint image intensity of the involved genes in the dChip visual representation. The correlation coefficients of 4 genes, identified as apoptosis-related, was examined in detail (Figure 5). IER3, IAPP, SST, and PHLDA2 all had good correlation between GeneChip and Q-PCR results. FGF9, a potential growth stimulating gene was also examined (Figure 6). Again, there was overall good correlation between the individual GeneChip and Q-PCR results.

**Table 9:** Correlation of GeneChip expression with quantitative RT-PCR.

Gene Symbol	Correlation	Probe Set	Exons	Gene Size (bp)	Fold Change (T/N)	P value GeneChip T vs. N
IER3	0.964	1237_at	1	1236	-4.6	0.0000
SST	0.925	37782_at	2	351	-12	0.0000
PHLDA2	0.909	40237_at	2	913	-2.01	0.0003
REG1B	0.875	35981_at	6	773	-499	0.0000
IAPP	0.823	37871_at	3	1462	-4.68	0.0033
REG1A	0.814	38646_s_at	6	808	-16.9	0.0000
FGF9	0.74	1616_at	3	1420	3.97	0.0031
CBLB	0.327	514_at	21	3923	3.01	0.0009
XPC	0.318	1873_at	16	3658	4.38	0.0018
HRK	0.273	34011_at	2	716	-2.27	0.0011
PTK2B	0.235	2009_at	38	4715	-2.94	0.0019
Average	0.655					

## Discussion

Whether there were degradative processes acting on the tissues prior to or during or after the extraction of the RNA can be guessed by the quality of the RNA. Each RNA specimen in this study was tested on an Affymetrix test chip, and each was found to be acceptable. Additional quality assessment was made by the dChip software. Only one specimen, a normal control, had Array Outliers greater than 5%, suggesting that it was subnormal (Table 2). However, since the percent outliers was only 5.94, the chip was included in the analysis.

Although, only solid tumor was utilized, there were undoubtedly a small percentage of blood, blood vessel, and connective tissue elements intermixed with the tumor tissue. Rarely, there might be a small amount of exocrine tissue. In the case of the normal islets used as controls, microscopic examination showed that greater than 90% of the tissue was islet. Any contaminants would probably have the effect of reducing the discriminant power to differentiate tumor from normal. Thus, t-test p values and fold changes would tend to under-represented and some, otherwise significant, genes might be missed. The actual data, represented by the hierarchical specimen clustering (Figure 3), showed strong differential gene expression relating to group identity as would be expected if the overall gene expression levels were accurate. All the normals clustered together, separate from all the tumors. Within the normals, the two male specimens clustered in one group, and the two female in another. All the normal islet preparations, which are composed predominantly of beta cells, clustered closer to the insulinoma tumors than to the other neuroendocrine tumor types. The gene clustering results revealed 19 apoptosis-related genes whose expression was suppressed in the islet tumors relative to the normals. This suggests that apoptosis may play a significant role in the development of these tumors.

One might have expected more variation in the gene expression levels in the tumors than in the normal islets, since tumors are often heterogonous. However the data on the average CV of the genes in the normal and tumor groups suggested that there was no more variation in the tumors (average CV of 30%) than in the normals (average CV of 31%). The low CV in the tumors may relate to the single mode of tumor formation (induction by the loss of the menin tumor suppressor). However, there was increased variation noted when the tumors and normals were combined (Figure 2). This was probably the result of the differences in expression between the tumors and the normals.

Of particular interest was the high proportion (3/8) of tumors expressing principally PP hormonal RNA. This was entirely consistent with pathological studies showing the preponderance of PP containing tumors in the pancreas of MEN1 patients [31]. The fact that the clinical classification of two patients (9 and 11) was different than indicated by the hormone expression profile of the tumor analyzed was a consequence of the facts that those patients had multiple tumors secreting multiple hormones but only insulin and gastrin and sometime PP over secretion are likely to result in a clinical diagnosis.

The use of the Students t-test for comparison of multiple genes might be questioned because the test was designed for comparison of only two groups. In this study, we confirmed that comparison of 923 genes would not generate an excess number of false positive results. Nevertheless, in the group of 193 genes finally selected at a p < = .005, we can expect that 1 of those genes is a false positive.

This study suggests that the overall effect of loss of function of menin is the suppression of gene expression. Nevertheless, there were 86 genes that were over-expressed in the tumors relative to the normals. Although we associate tumorigenesis with increased rates of growth, only two of eleven Cell Cycle and Cell Proliferation genes were increased in the tumors. Since tumor growth may also be significantly affected by rates of cell death, it is perhaps significant that there were no Cell Death genes significantly increased in the tumors relative to the controls.

The correlation of GeneChip results with quantitative real-time PCR (Q-PCR, Table 9) was relatively good. However, there were some genes that correlated poorly (correlation coefficient less than 0.6). Interestingly, most of the genes with poor correlation coefficients had a large number of exons, whereas those with high correlation coefficients had a low number of exons. Since exhaustive testing of alternative primer pairs for Q-PCR was not made, it is possible that correlation coefficients of some genes could be improved by the use of other primers.

Four studies of global gene expression in pancreatic islets have been published recently [32-35]. Cardozo et al [32] have used microarrays to look for NF-kB dependent genes in primary cultures of rat pancreatic islets. Shalev et al [33] have measured global gene expression in purified human islets in tissue culture under high and low glucose concentrations. They noted that the TGFβ superfamily member PDF was down regulated 10-fold in the presence of glucose, whereas other TGFβ superfamily members were up regulated. In the current study, none of the TGFβ superfamily members were significantly different between tumor and normal. Scearce et al [34] have used a pancreas-specific micro-chip, the PanChip to analyze gene expression patterns in E14 to adult mice. Only a few specific genes were noted in the paper, and none of them had human homologs of significance to the current study. Maitra et al [35] conducted a study which in many ways was similar to the current one. They compared gene expression, using the Affymetrix U133A chip, in a series of sporadic pancreatic endocrine tumors with isolated normal islets. There was no overlap in the genes they identified (having a three-fold or greater difference in expression) with the genes we identified (having a two-fold or greater difference in expression). This is quite surprising, but perhaps suggests that sporadically arising tumors may have a quite different pattern of gene expression than tumors arising as a result of menin loss or dysfunction. Another possible cause of the differences may be the different Affymetrix GeneChips used in the two studies.

The question of which (if any) of the genes delineated in this study are a direct and necessary affect of loss-of-menin tumorigenesis cannot be determined by this study alone. Firstly, the activity of many genes are regulated both by their levels of expression and by post-translation modifications, such as phosphorylation. Secondly, the microchips used in this study represent only about 1/3 of the total number of human genes. Thirdly, it is not certain that the initiating gene changes caused by loss-of-menin are persistent in the tumors that develop. However, there were some genes, which because of their association with growth or apoptosis are of special interest. The general suppression of apoptosis related genes noted in this study (Figure 4) has been highlighted by the recent study of Schnepp *et al*, [36] who showed a loss of menin suppression of apoptosis in murine embryonic fibroblasts through a caspase-8 mechanism. Specific apoptosis-related genes which were suppressed in the tumors in the current study, and which were confirmed by Q-PCR include IER3, SST, PHLDA2, and IAPP. IER3 (IEX-1) is regulated by several transcription factors and may have positive or negative effects upon cell growth and apoptosis depending upon the cell-specific context [37]. Several studies have shown that it can be a promoter of apoptosis [38-40]. Somatostatin has shown a wide range of growth inhibitory activity *in vitro *and *in vivo *[41-57].PHLDA2 (TSSC3) is an imprinted gene homologous to the murineTDAG51 apoptosis-related gene [58], and may be involved in human brain tumors [59]. IAPP (amylin) is a gene which has contrasting activities and has been associated with experimental diabetes in rodents [60]. Amylin deposits were increased in islets of patients with gastrectomy-induced islet atrophy [61]. On the other hand, exposure of rat embryonic islets to amylin results in beta cell proliferation [62]. In contrast, amylin has been shown to induce apoptosis in rat and human insulinoma cells *in vitro *[63,64]. In contrast to the suppression of apoptosis-related genes, FGF9 (Figure 6), a growth promoting gene, was significantly increased in the neuroendocrine tumors. This protein has been reported to play roles in glial cell growth [65], chondrocyte growth [66], prostate growth [67], endometrial growth [68], and has been suggested to have a role in human oncogenesis [69].

A recent report by Busygina et al [70] suggested that loss of menin can lead to hypermutability in a *Drosophila *model for MEN1. The spectrum of mutation sensitivity suggested that there was a defect in nucleotide excision repair. Whether the defect was a direct or indirect effect of menin loss was not stated. In the current study, there was a 2.44-fold decrease, in the tumors, in the expression of ERCC4 (Table 5), a gene involved in nucleotide excision repair. In addition, XRCC4, a gene involved in double-strand break repair, was also decreased in the tumors in the current study.

## Conclusion

This first study of global gene expression in neuroendocrine tumors arising in the pancreas of patients with the MEN1 syndrome has identified many genes that are differentially expressed, as compared with normal human islet cells. A number of these genes are strongly over/under expressed and are attractive candidates for further investigation into the mechanisms by which menin loss causes tumors in pancreatic islets. Of particular interest was a group of 24 apoptosis-related genes that were significantly differentially expressed (mostly underexpressed) in the group of neuroendocrine tumors. The underexpression of these apoptosis-related genes may be related to neoplastic development or progression in these MEN1-related neuroendocrine tumors.

## Methods

### Human Tissue Specimens

Tumor specimens were obtained from patients with the MEN1 syndrome who had undergone surgery for islet-cell tumors of the pancreas. The specific germline mutations in the menin tumor suppressor gene were identified and previously reported [6] for each of the patients. Six of the patients had frame-shift mutations and one had a nonsense mutation. Informed consent was obtained in advance, and tumor tissues not needed for pathological analysis were snap frozen in liquid nitrogen, and kept frozen at -70° prior to RNA extraction. Normal pancreatic islets (which were originally intended for human transplatation studies, but were not used) were isolated from brain-dead donors by a collagenase procedure, as previously described [71], and were then frozen until used for extraction of RNA. Human Studies Committee approval from Washington University School of Medicine was obtained for this study.

### Isolation of RNA from Tissue Specimens

Approximately 50 mg of tissue was removed from each frozen tumor specimen and homogenized with a mortar and pestle (Qiagen, Qiashredder Kit), and RNA was extracted using the Rneasy Mini Kit (Qiagen, Inc.), and quantified by UV absorbance. RNA was similarly isolated from the normal human islet preparations.

### GeneChip Hybridization and Analysis

The RNA was submitted to the GeneChip facility of the Siteman Cancer Center at Washington University School of Medicine. There, biotin labeled cRNA was prepared and hybridized to U95Av2 microarray chips (Affymetrix). The fluorescence of individual spots was then measured and the data returned on compact discs. We analyzed the gene expression data and made comparisons between groups using the dChip computer program [30]. Following normalization (to equalize the overall intensity of each chip), the expression of each gene was determined by statistical modeling procedure in the dChip software. Each gene was represented by an array of 10 perfect match oligonucleotide spots and 10 mismatch oligonucleotide spots on the U95Av2 chip. The dChip program examines all the spots on all the chips involved in the study, and by a statistical procedure determines single and array outliers. These outliers can be considered as "bad" readings, and removed from further consideration.

### Quantitative RT-PCR

The same preparations of total RNA that were used to probe the GeneChips were also used to prepare c-DNA for quantitative RT-PCR analysis of gene expression. C-DNA was first prepared using Superscript II reverse transcriptase (Invitrogen, Inc.). Primers for each gene were designed to produce products of 100 to 150 bp that spanned exon boundaries (when possible). The primer pairs are shown in table 10.

**Table 10:** 

**Gene**	**Forward Primer**	**Reverse Primer**
CBLB	cacgtctaaatctatagcagccagaac	tgcactcccaagcctcttctc
FGF9	cggcaccagaaattcacaca	aaattgtctttgtcaactttggcttag
HRK	agctggttcccgttttcca	cagtcccattctgtgtttctacgat
IAPP	ctgctttgtatccatgagggttt	gaggtttgctgaaagccacttaa
ER3	ccagcatctcaactccgtctgt	caccctaaaggcgacttcaaga
SST	cccagactccgtcagtttctg	tacttggccagttcctgcttc
PHLDA2	tgcccattgcaaataaatcact	ctgcccgcccattcct
PTK2B	gtgaggagtgcaagaggcagat	gccagattggccagaacct
REG1A	cctcaagcacaggattccaga	acatgtattttccagctgcctcta
REG1B	gggtccctggtctcctacaagt	catttcttgaatcctgagcatgaa
XPC	gcccgcaagctggacat	atcagtcacgggatgggagta

The Sybr Green technique on an Applied Biosystems model GeneAmp 5700 instrument was utilized. Relative quantitation using a standard c-DNA preparation from an *in vitro *islet tumor cell line was utilized.

## Competing interests

The author(s) declare that they have no competing interests.

## Authors' contributions

All authors contributed equally to this manuscript.
